# Rare consecutive femoral lesser trochanteric avulsion in an adolescent following sporting activities; a case report

**DOI:** 10.1016/j.radcr.2022.10.077

**Published:** 2022-11-26

**Authors:** John Ifeanyi Ubimago, Meraiyebu Aminyene Essien, Kingsley Iyoko Iseko, Omachoko Emmanuel Oguche, Nkechi Gift Inyang, Kalma Pembari Sabiya

**Affiliations:** a Department of Family Medicine, Limi Hospital, Abuja, Nigeria; b Department of Radiology, Limi Hospital, Abuja; c Department of Orthopedics, University of Abuja Teaching Hospital, Nigeria

**Keywords:** Avulsion, Lesser, Trochanter, Femur, Adolescent, Conservative

## Abstract

Avulsion of the lesser trochanter is a rare but disturbing condition, which usually occurs in males between the ages of 7-16 years with significant physical activities or in athletes. Diagnosis is more often than not challenging, but with a good history, physical examination, and imaging modality, diagnosis can be clinched, and the prognosis is good even with a conservative management approach. This case report is that of a 12-year-old male who suddenly fell while participating in sporting activities in school. As a consequence of the fall, he felt severe pain in the left groin region with an associated inability to bear weight on the affected limb. A radiograph study of the hip revealed a fracture of the left lesser trochanter. Based on the diagnosis, a conservative approach, which entails the use of analgesics, and partial weight-bearing mobilization with axillary crutches to take the weight off the affected limb for a period, was the choice of management for this subject. Fifteen weeks following the conservative management for the avulsion of the left femoral lesser trochanter fracture, the subject sustained a similar injury to the contralateral groin, consequential to return to sporting activities. In conclusion, rare as avulsion of the lesser trochanter may be, a high index of suspicion must be raised in any adolescent with a painful limp following engagement in any sporting activities, and such individuals should have a radiograph study done to achieve prompt and effective care.

## Introduction

The lesser trochanter is a growth region type apophysis, which is attached to the iliopsoas muscle. Avulsion fractures in this region are generally secondary to strenuous physical activities [1]. This usually occurs in males between the ages of 7 to 16 years old, with a peak of incidence at age 14. Its incidence is low; however, with the increasing participation in athletics at an early age in high-demand competitions, this kind of trauma has become more common.

As is the case of this 12-year-old male which is being reported. He was noticed to have pain in the left groin with an associated difficulty walking following a fall during sporting activities.

The signs and symptoms that may occur include pain in the groin region, difficulty walking, and limitation of hip flexion and adduction. The definitive diagnosis is made with a plain radiograph in the anteroposterior view. In imaging, you may find a fracture fragment pulled medially and proximally by iliopsoas tendon insertion into the fragment [2].

In children, where the apophysis is still mainly cartilaginous and not obvious on x-rays, ultrasound scans, or MRIs, and sometimes a good index of suspicion is what is needed to make a diagnosis.

Avulsion of the lesser trochanter during sporting events is a rare occurrence and may remain concealed in a child. Emergency department diagnosis of this entity is based on history and physical examination and remains a challenge and a daunting task because of the rarity of this condition.

## Case report

A 12-year-old male presented at the emergency department of The Limi Multispecialty Hospital, Abuja, 1 hour following a fall while participating in a sprint sporting event in school. The complaints were chiefly; that of acute pain in the left hip region and inability to bear weight on the affected limb.

For femoral fractures of the trochanteric region in children and adolescents, only two mechanisms have been identified to cause a fracture of the proximal femur; high-energy trauma or predisposing bone pathologies with inadequate trauma (eg, simple fall, movement).The mechanism of injury, in this case, was likely due to trauma to the hip following impact when the subject fell to the ground.

**Fig. 1:**
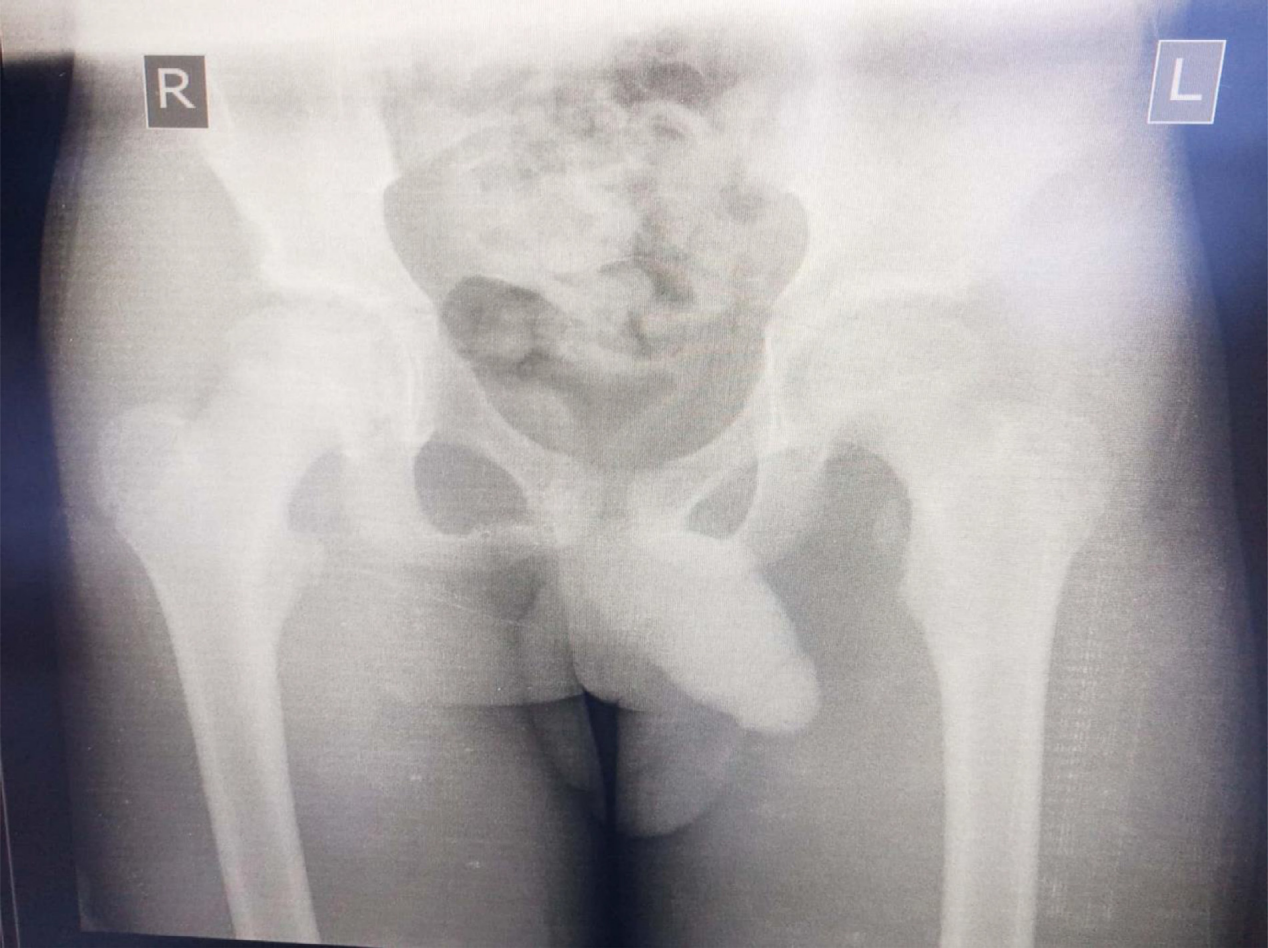
Radiograph of the pelvis of a 12-year-old patient showing an avulsion of the left lesser trochanter (arrow) compared to the right normal hip.

The physical examination revealed a healthy child with an antalgic gait. There was no obvious deformity, swelling, or ecchymosis; however, there was marked tenderness at the anteromedial aspect of the left hip/groin. There was no limitation of passive movement around the affected hip, but active flexion was painful with some degree of weakness especially, with flexion greater than 90 degrees around the affected hip. No neurological deficits were detected. The peripheral pulses were present and symmetrical in both legs. The left hip and pelvic radiographs revealed a single fragment avulsion of the lesser trochanter with about 1 centimeter of upward displacement (Fig. 1). A conservative therapeutic approach which entails, the use of analgesics and partial weight-bearing with axillary crutches in the contralateral hand was instituted. Full non-weight bearing ambulation could not be instituted in this patient due to the co-existing distal radius fracture on the ipsilateral forearm which was treated nonoperatively also in a Colle's cast.

**Fig. 2:**
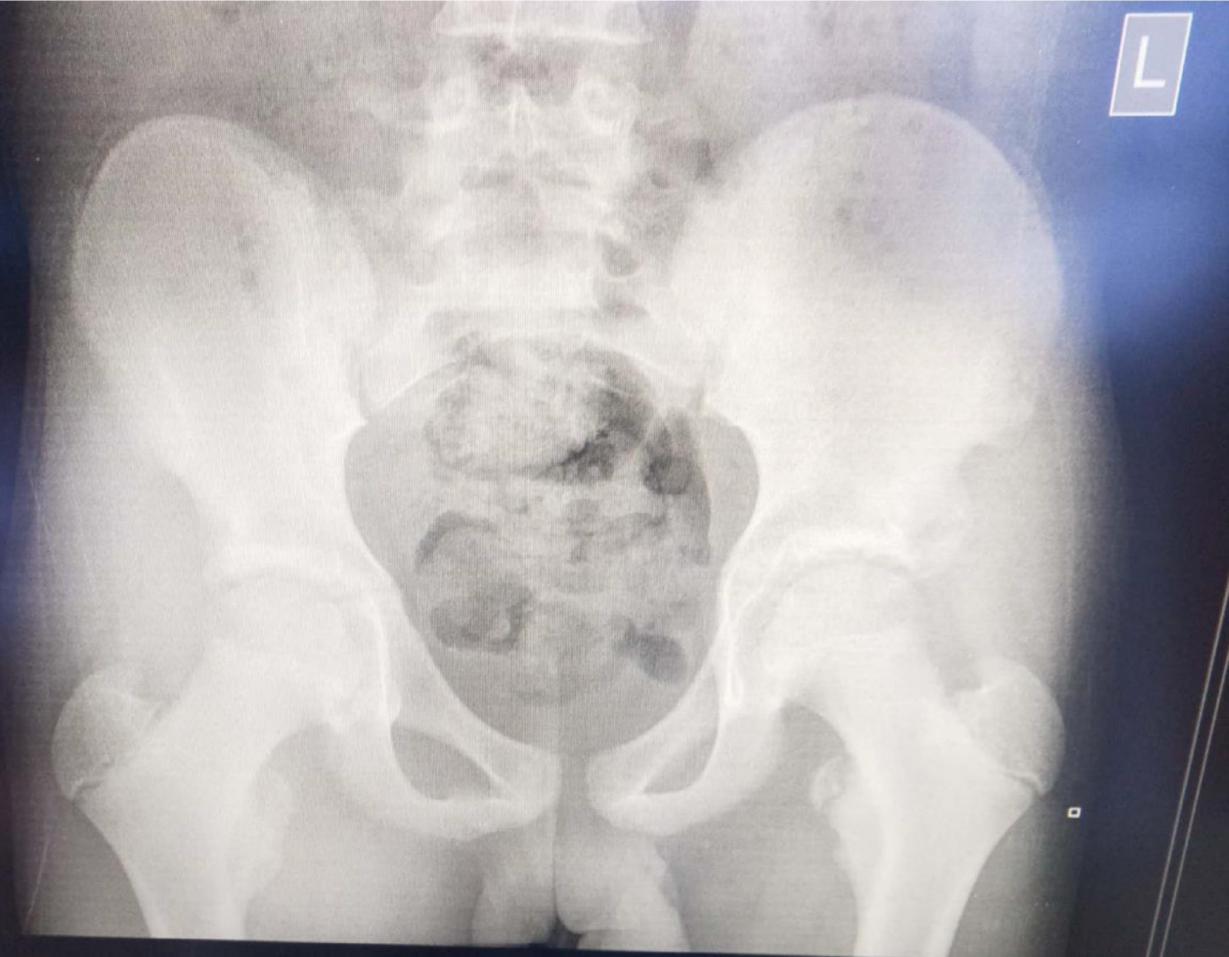
Radiograph of the pelvis 9 weeks following conservative and nonsurgical management of left lesser trochanter avulsion injury.

The subject was followed-up by an orthopedic surgeon in the clinic for about 9 weeks while undergoing the conservative and nonsurgical approach to management. In his latest clinic visit, he was found to have made a remarkable improvement and has since returned to normal function. See Figure 2 for a radiograph of the pelvis after 9 weeks of conservative management. It shows evidence of good callus formation around the site of the injury. With this case report, it is clear that lesser trochanter avulsion injuries in adolescent athletes can be treated conservatively with an average return to normal function and sports time of fewer than three months.

**Fig. 3:**
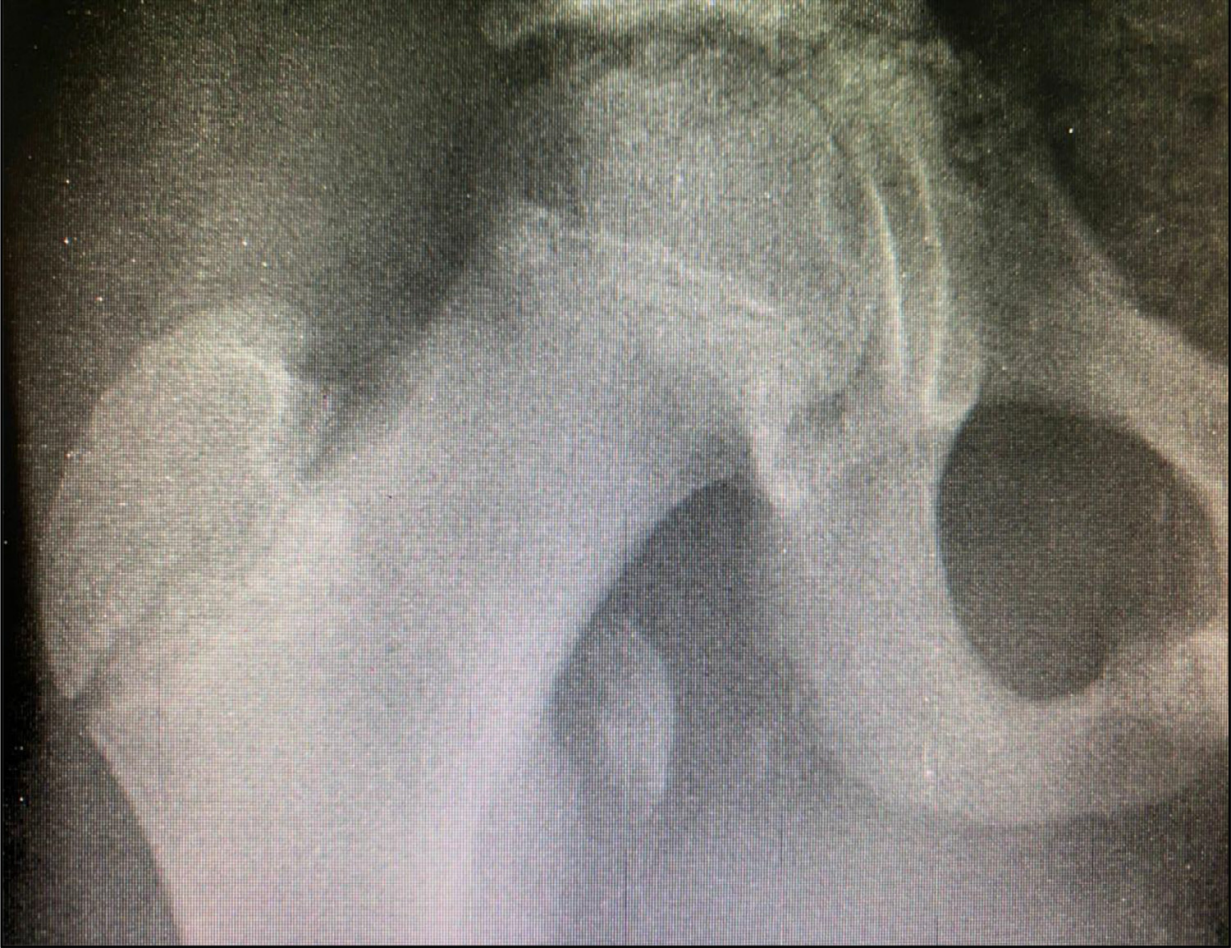
Radiograph of the right hip of a 12-year-old patient showing an avulsion of the right lesser trochanter 15 weeks after conservative management of similar injury to the left lesser trochanter.

Fifteen weeks following the conservative management for the avulsion of the left femoral lesser trochanter fracture, the patient had significant improvement and returned to his usual active lifestyle, including engaging in sporting activities. While trying to kick a football, he felt a sudden pain and pull at the right groin. Subsequently, he was not able to move the limb or bear weight on the affected limb. This prompted his presentation at the outpatient department, where the radiograph study revealed a fracture of the right femoral lesser trochanter (Fig. 3). A conservative management approach was still the choice for this injury.

However, further history was taken to ascertain the presence of other risk factors that might make our subject fracture-prone. Given the consecutive bilateral lesser trochanteric fractures and fracture to the distal radius (Colles fracture) of the ipsilateral forearm. The subject was found to be well fed, as evidenced by his weight and height, which were 42 kg and 150 cm, respectively, with a body mass index (BMI) of 18.7 Kg/M^2^, which was adjudged to be appropriate for his age and sex (56% weight-for-age percentile and 55% height-for-age percentile). There was no history suggestive of rickets, osteogenesis imperfecta, transient synovitis of the hip, osteosarcoma, or Perthes disease in our subject.

## Discussion

Isolated fractures of the lesser trochanter are unusual injuries in adolescence, accounting for only 0.3%t of proximal femur fractures. Avulsion fractures generally occur during adolescence. They are usually seen in children between the ages of 7-16 years, but most commonly occur at the age of 14.

In a study done by Thomas Ruffing et al., an avulsion fracture of the lesser trochanter was diagnosed in 4 boys and 1 girl. The mean age of the patients was 13.8 years (range: 13-15 years). They observed 2 type II and 3 type III fractures. The patients received similar non-operative treatment. Follow-up was performed at a mean of 4.9 years (range: 3.5-6.2 years) after injury. All patients returned to competitive sports. The Harris Hip Score was 100 of 100 points. History and provocation test concerning an ischio-femoral impingement was negative in all patients.

In another study by Volpi A. et al., avulsion fractures of the lesser trochanter were found in 30 patients (26 male, 4 female). The average age at the time of injury was 14.2 years (range: 7.7-17.5 years) with an average body mass index of 21.1 (range: 16.9-29.7). The mean follow-up time was 102 days. The injuries were traumatic and sustained during sports in all 30 patients. The sports subjects were involved in before the injury include hockey, soccer, basketball, baseball, track and field, football, and cheerleading.

Avulsion of the lesser trochanter usually occurs from an acute injury most commonly related to sporting events. The main cause of such avulsions is a forceful contraction of the iliopsoas tendon during hip flexion. The excessive stress concentrated at the site leads to a tensile failure of the apophysis of the lesser trochanter [3].

The diagnosis may be inferred by the patient's age and mechanism of injury. Nevertheless, other etiologies of pediatric hip pathology, including septic arthritis, slipped capital femoral epiphysis, osteosarcoma, and Perthes’ disease, must be ruled out in this age group.

The most frequent presenting clinical scenario is groin pain and limp, with little external evidence of trauma. The physical examination often reveals tenderness over the medial aspect of the hip and pain with hip flexion greater than 90° (Ludloff sign). The diagnosis is confirmed most times with radiography. The avulsed piece is most often times displaced proximally by the pull of the iliopsoas tendon.

Symptomatic treatment is proposed, with limited weight-bearing on crutches for 3-4 weeks and analgesia as required. Complete healing can take up to 2 months, and sports should be avoided during this time.

Surgical intervention is indicated where a non-union or fibrous union has occurred, resulting in chronic pain with motion at the fracture site. If the avulsed fragment is displaced more than 3 centimeters, surgical reattachment or excision is advised [3].

Nevertheless, a study by Fernbarch and Wilkonson demonstrated that operative treatment is rarely indicated. This study looked at 20 male adolescents engaged in competitive sports. Results of those treated conservatively were comparable to open reduction and internal fixation of the fragment, regardless of the degree of displacement. The majority of patients with this type of injury eventually become asymptomatic and can return to their original activity levels. This is valid even in the setting of persistent radiographic abnormalities.

## Recommendations

Initial non–weight-bearing ambulation is advocated and after significant pain control is achieved, the progressive partial load should be introduced. After 45 days, the subject is expected to be asymptomatic and could walk without support. In 90 days, the subject should be free to return to his sports practices.

## Conclusion

In subjects who are within the age group of 7- 16 years with a painful limp after sporting activities, there should be a high index of suspicion for avulsion fractures. Radiographs should be done, and other pathologies excluded. Symptomatic treatment is recommended with a gradual return to sports after at least 3 months.

## Patient consent

We attest that a duly signed consent form has been administered to the father of the patient who is a minor with detailed informed explanation and signed consent obtained to use the case for our case report.
